# Exposure and survival of wild raptors during the 2022–2023 highly pathogenic influenza a virus outbreak

**DOI:** 10.1038/s41598-025-90806-6

**Published:** 2025-02-24

**Authors:** Kelsey M. Rayment, Dana Franzen-Klein, Elizabeth Kurimo-Beechuk, Rebecca L. Poulson, Justin Brown, Kristelle Mendoza, Matthew Etterson, Frank Nicoletti, Carol Cardona, David E. Stallknecht, Victoria Hall

**Affiliations:** 1 The Raptor Center, College of Veterinary Medicine, University of Minnesota, St. Paul, MN USA; 2 Southeastern Cooperative Wildlife Disease Study, Department of Population Health, College of Veterinary Medicine, University of Georgia, Athens, GA USA; 3 Department of Veterinary and Biomedical Sciences, College of Agricultural Sciences, The Pennsylvania State University, University Park, PA USA; 4 Department of Veterinary and Biomedical Sciences, College of Veterinary Medicine, University of Minnesota, St. Paul, MN USA; 5 Hawk Ridge Bird Observatory, Duluth, MN USA; 6 Swenson College of Science and Engineering, University of Minnesota – Duluth, Duluth, MN USA; 7 Present Address: One Health Institute, School of Veterinary Medicine, University of California, Davis, Davis, CA USA

**Keywords:** Highly pathogenic avian influenza, Raptors, Serology, Wildlife, Wildlife rehabilitation, Influenza virus, Influenza virus, Ecology

## Abstract

The global outbreak of clade 2.3.4.4b H5N1 highly pathogenic influenza A virus (HP H5N1) has had an unprecedented impact on wild birds including raptors, but long-term population impacts have not been addressed. To determine if raptors survive infections with HP H5N1, raptors from the upper Midwest United States were serologically tested for antibodies to influenza A virus (IAV), H5 and N1. Raptors were sampled at The Raptor Center’s (University of Minnesota) wildlife rehabilitation hospital and at Hawk Ridge Bird Observatory. Samples were tested for IAV antibodies using a commercially available blocking ELISA, with positive samples tested for antibodies to H5 and N1. Antibodies to IAV were detected in 86 out of 316 individuals representing 7 species. Antibodies to H5 and N1 were detected in 60 individuals representing 6 species. Bald eagles had the highest seroprevalence with 67/97 (69.1%) seropositive for IAV and 52 of these 67 (77.6%) testing positive for antibodies to both H5 and N1. Prevalence of antibodies to IAV observed in this study was higher than reported from raptors sampled in this same region in 2012. The high prevalence of antibodies to H5 and N1 indicates a higher survival rate post-HP H5N1 infection in raptors than previously believed.

## Introduction

Influenza A viruses (IAV) represent a threat to wildlife, agriculture, and humans across the globe^1,2^. The threat of these viruses has increased significantly since the emergence of the highly pathogenic (HP) (A/goose/Guangdong/1/1996) (Gs/GD) lineage of IAV in 1997^1,3^. The current global outbreak of clade 2.3.4.4b Gs/GD HP IAV (H5Nx) has had an unprecedented impact on wild birds globally^1,4,5^. In North America, clade 2.3.4.4b HP IAV H5N1 (HP H5N1) was first detected in late 2021 and rapidly spread through migratory flyways^6–8^. It was first documented in Minnesota and the upper Midwest in late March 2022^5,9^. In North American wild birds, there have been large scale mortality events with more than 9,300 reported infections in over 190 species and sustained transmission continuing over multiple waterfowl migration seasons^5,10^. Reported wild bird detections are likely a significant underestimate of the impact on wild bird populations, as not every individual in a mass die off is tested and reported, and not all wild birds that succumb to this virus are found or tested^11^.

Raptors are highly susceptible to infection with HP H5N1 and often develop severe clinical disease^12–15^. Previous serosurveys focused on wild and captive raptor populations have found low antibody detection rates to IAVs^16–18^. A 2019 study in Norway did not detect antibodies to IAV in nestling white-tailed eagles (*Haliaeetus albicilla*) or northern goshawks (*Accipiter gentilis*)^17^. A study conducted in the United Arab Emirates between 2003 to 2006 screened for H5, H7 and H9 antibodies in 592 captive falcons used for falconry; one falcon had antibodies to H5 (0.2%) and 78 had antibodies to H9 (13.2%)^18^. A 2012 serosurvey conducted at The Raptor Center (TRC) at the University of Minnesota tested several wild raptor species that were admitted for rehabilitation^16^. This study reported that 5.1% of bald eagles (*Haliaeetus leucocephalus*) tested positive for IAV antibodies; additional tests for subtype specific antibodies were not performed^16^. Limitations of these previous studies include low sample numbers, limited species and spatial representation, and inclusion of captive animals. HP H5N1 also was not present in these sampled populations in North America when this research was conducted.

Wildlife rehabilitation centers are well positioned to conduct surveillance of wildlife pathogens such as HP H5N1, as they receive a large number of animals from a wide geographic area and are located at the human-wildlife interface^19,20^. Bird banding stations provide another opportunity to collect disease surveillance samples from apparently healthy wild birds and have been routinely used to monitor the health of wild bird populations.

The goal of this study was to use serological testing to determine the prevalence of antibodies to IAV, H5, and N1 in multiple wild raptor species from the upper Midwest following the 2021 introduction of HP H5N1 to North America. Samples were collected during the fall 2022 and spring 2023 migration seasons, which immediately followed the peak of disease transmission in the region during the 2022 spring migration.

## Results

For the subset of individuals tested by blocking enzyme-linked immunosorbent assay (bELISA) that had both serum and plasma samples collected at the same time, the average plasma absorbance was subtracted from the average serum absorbance. The average difference in the absorbance values was 0.019, the median difference was −0.008, and the range of the difference was −0.035 to 0.387 (n = 73). A significant difference in absorbance between plasma and serum was not detected (p < 0.05) using a paired sample T test.

During the study period of September 13, 2022, through April 27, 2023, TRC admitted 394 wild raptors representing 22 species that tested negative for HP H5Nx using real time reverse transcriptase polymerase chain reaction (RT-PCR); serology samples were obtained from 316 (80%) of these individuals representing 21 species (Table 1). Samples were obtained from 0 –155 days post-admission (median = 0 days, mean = 4.5 days), with 92% of birds sampled within ten days of admission. During the fall 2022 banding period at HRBO, 1782 individuals were banded representing 16 species. Serum samples were obtained opportunistically from 51 (2.9%) individuals, which represented seven different species (Table 1). In total, 367 individuals were screened for IAV NP antibodies using the bELISA; antibodies were detected in 86 (23%) individuals representing 7 species (Table 1).

**Table 1:** Prevalence of avian influenza virus antibodies as determined by a commercially available bELISA in raptors presented to The Raptor Center (St. Paul, MN) and captured at Hawk Ridge Bird Observatory (Duluth, MN).

Species	Number positive	Number tested	Percent positive
**The Raptor Center**			
American Kestrel (*Falco sparverius*)	0	2	0
Bald Eagle (*Haliaeetus leucocephalus*)	67	97	69.07
Barred Owl (*Strix varia*)	4	57	7.02
Broad-winged Hawk (*Buteo platypterus*)	0	10	0
Cooper’s Hawk (*Accipiter cooperii*)	0	10	0
Eastern Screech-owl (*Megascops asio*)	0	9	0
Great Horned Owl (*Bubo virginianus*)	5	36	13.89
Long-eared Owl (*Asio otus*)	0	1	0
Merlin (*Falco columbarius*)	0	5	0
American Goshawk (*Accipiter atricapillus*)	0	2	0
Northern Harrier (*Circus hudsonius)*	0	2	0
Northern Saw-whet Owl (*Aegolius acadicus*)	0	6	0
Osprey (*Pandion haliaetus*)	0	3	0
Peregrine Falcon (*Falco peregrinus*)	0	2	0
Rough-legged Hawk (*Buteo lagopus*)	1	4	25
Red-shouldered Hawk (*Buteo lineatus*)	0	1	0
Red-tailed Hawk (*Buteo jamaicensis*)	6	53	11.32
Short-eared Owl (*Asio flammeus)*	0	1	0
Snowy Owl (*Bubo scandiacus*)	0	1	0
Sharp-shinned Hawk (*Accipiter striatus*)	0	6	0
Turkey Vulture (*Cathartes aura*)	1	8	12.5
**Total**	84	316	26.58
**Hawk Ridge Bird Observatory**			
Cooper’s Hawk (*Accipiter cooperii*)	0	2	0
Long-eared Owl (*Asio otus*)	0	1	0
American Goshawk (*Accipiter atricapillus*)	1	14	7.14
Northern Harrier (*Circus hudsonius)*	0	3	0
Northern Saw-whet Owl (*Aegolius acadicus*)	0	3	0
Red-tailed Hawk (*Buteo jamaicensis*)	1	9	11.11
Sharp-shinned Hawk (*Accipiter striatus*)	0	19	0
**Total**	2	51	3.92

For samples that were positive on the initial bELISA screen, 60 out of 86 (69.8%) had antibodies specific to both H5 and N1 (Table 2). Bald eagles represented the largest proportion (67/86; 77.9%) of the bELISA positive samples and most of those had antibodies to H5 and N1 (52/67; 77.6%). Of the other species that had IAV NP antibodies, all but the American Goshawk (*Accipiter atricapillus*) had at least one individual with antibodies to H5 and N1 (Table 2).

**Table 2:** Antibodies to H5 and N1 detected in IAV positive sera and plasma samples collected from The Raptor Center (St. Paul, MN) and Hawk Ridge Bird Observatory (Duluth, MN).

Species	bELISA positive no. individuals	H5N1 positive no. individuals	H5N1% positive	H5 only positive	N1 only positive	non H5N1
American Goshawk (*Accipiter atricapillus*)	1	0	0.00	0	0	1
Bald Eagle (*Haliaeetus leucocephalus*)	67	52	77.61	3	6	6
Barred Owl (*Strix varia*)	4	1	25.00	1	0	2
Great Horned Owl (*Bubo virginianus*)	5	2	40.00	2	1	0
Rough-legged Hawk (*Buteo lagopus*)	1	1	100.00	0	0	0
Red-tailed Hawk (*Buteo jamaicensis*)	7	3	42.86	4	0	0
Turkey Vulture (*Cathartes aura*)	1	1	100.00	0	0	0
**Total**	**86**	**60**	**69.77**	**10**	**7**	**9**

The bald eagle (n = 97) was the most frequently sampled species at TRC. The majority of bald eagles sampled were classified as adults (61/97, 62.9%), followed by sub-adults (26/97, 26.8%), then juveniles (10/97, 10.3%). In total, 69.1% (67/97) of all bald eagles sampled were positive for IAV NP antibodies. Adults (48/61, 78.7%) had the highest seroprevalence, followed by sub-adults (18/26, 69.2%) (Table 3). Antibodies specific to H5 and N1 were detected in adult and sub-adult eagles but not juvenile eagles (Table 3).

**Table 3:** Age breakdown and subtyping of IAV positive bald eagles sampled at The Raptor Center (St. Paul, MN).

Age	H5 only positive	H5N1 positive	N1 only positive	non H5N1	Number bELISA positive	% bELISA positive	Number tested
Adult	3	37	3	5	48	78.69	61
Sub-adult	0	15	3	0	18	69.23	26
Juvenile	0	0	0	1	1	10.00	10
**Total**	**3**	**52**	**6**	**6**	**67**	**69.07**	**97**

**Fig. 1:**
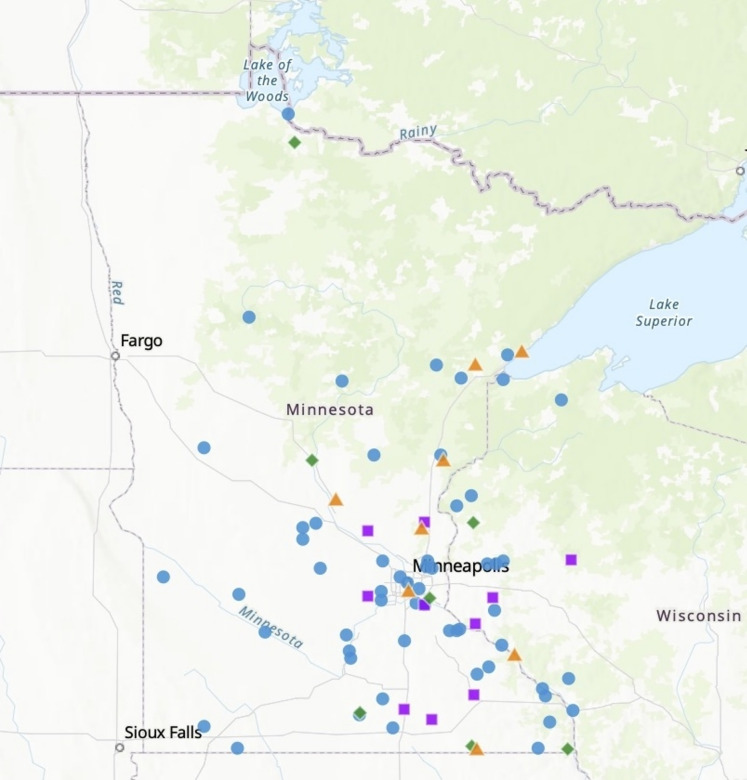
Recovery location of IAV antibody positive birds sampled at The Raptor Center, St. Paul, MN. IAV antibody status was determined with a bELISA screen. Subtyping of antibodies to H5 determined by hemagglutinin inhibition (HI) and virus neutralization (VN). Specificity of antibodies specific to N1 determined by enzyme linked lectin binding assay (ELLA). Blue circles represent individuals with antibodies to H5N1. All points represent the GPS coordinates of the recovery address as reported by the individual who found the bird in the field. Purple squares represent individuals with antibodies to H5 only. Green diamonds represent individuals with antibodies to N1 only and orange triangles represent individuals with antibodies non-H5N1 variants.

Raptors sampled at TRC were admitted from Minnesota (n = 279), North Dakota (n = 5) and Wisconsin (n = 32). Birds which tested positive to H5 and N1 antibodies detected in this study were found in Minnesota and (n = 69) Wisconsin (n = 15) (Fig. 1).

## Discussion

The results of this study provide evidence that raptor species in the upper Midwest, United States have been infected with and survived infection with HP H5N1. The three most represented species that tested positive for antibodies to H5 and N1 included: bald eagles (65/93, 69.9%), red-tailed hawks (*Buteo jamaicensis*) (7/57, 12.3%), and great horned owls (*Bubo virginianus*) (5/34, 14.7%). These three species also had the highest prevalence of RT-PCR positive test results at the time of admission during active patient surveillance conducted at TRC in 2022, during the height of transmission of the virus^9^. Bald eagles had the highest H5 and N1 seropositivity compared to all other species tested. This could be due to a multitude of factors including the impact of natural history and diet on exposure risk, and differences in disease susceptibility between species^9,13,21,22^. Other species that tested seropositive to IAV subtypes H5 and N1 include rough-legged hawks (*Buteo lagopus*), red-tailed hawks, turkey vultures (*Cathartes aura*), and great horned owls, all of which have been reported to feed on carrion or hunt wild waterfowl which are likely routes of exposure^22^. Some of the species that tested seropositive in this study included very few representative individuals, which precludes the ability to draw any conclusions about the regional population. For example, seroprevalence in rough-legged hawks was 25%, but only four individuals were tested and only one bird tested positive for antibodies to H5 /N1.

The results of this study are particularly interesting when compared to a similar study conducted at TRC in 2012 that assessed the seroprevalence of IAV NP antibodies in six species of raptors^16^. This prior study used the same initial screening test and was sampling from the same wild raptor population that is included in this study. At the time of the 2012 study, the Gs/GD lineage of HP IAV (H5Nx) had not yet been detected in North America, as the first introduction occurred in November 2014^1^. Bald eagles in this study were found to have the highest prevalence for IAV NP antibodies at 5.1% (22/406). A single seropositive individual was found in the following species: great horned owl and Cooper’s hawk (*Accipiter cooperii*). The results of the 2022–2023 serosurveillance presented here show a marked increase in IAV NP seroprevalence in bald eagles 69.9% (65/93) and in other raptor species, which correlates with the ongoing HP H5N1 outbreak at the time of sample collection. The landscape of IAV in wild birds has changed significantly in the past 10 years highlighting the impact, rapid spread, and changes in HP H5N1 risk to species with previously low rates of IAV exposure.

The species most frequently sampled at TRC in this study was the bald eagle. The vast majority of the bald eagles which had IAV NP antibodies were aged as greater than one year at the time of admission (66/67, 98.5%). Additionally, the single juvenile with IAV NP antibodies did not have antibodies specific to H5 or N1. The older age classes could potentially have had existing immunity prior to the start of the outbreak, therefore making it more likely that they would survive compared to naïve younger birds; however, based on the previous low prevalence of antibodies in this species, a significant impact between age classes would not be expected. Differences in IAV seroprevalence between age classes has been documented in other species^23,24^. The difference in seroprevalence between adults and younger birds may have implications in disease epidemiology over time. As HP H5N1 continues to circulate over multiple breeding seasons, it is possible that there will be different impacts on different age classes, potentially impacting population structure and recruitment^13^. Further research is needed to evaluate serology trends related to age and disease prevalence in the environment to learn more about the behavior of this disease in wild raptors.

One of the limitations of surveillance conducted at wildlife rehabilitation centers is the bias towards unhealthy or injured wildlife^20^. To help address this bias, samples were collected from apparently healthy migrating raptors at Hawk Ridge Bird Observatory (HRBO) to supplement the samples obtained from rehabilitation patients. Wildlife rehabilitation center populations are also biased towards animals living near humans, as that increases the probability of them being found and brought into care^20^. Despite these limitations, there are benefits to sampling from a wildlife rehabilitation center. Wildlife rehabilitation centers may admit animals from a wide geographic region, which may not be able to be mimicked with targeted trapping in a few locations. This advantage is highlighted well in this study, as samples were obtained from birds recovered across the entire state of Minnesota as well as surrounding states. In addition, wildlife rehabilitation centers can collect samples from species that are notoriously hard to capture, handle and sample. This is especially relevant when it comes to adult raptors which are naturally more dispersed on the landscape and do not typically congregate in large groups during migration. Additionally, viral genotypes of HP H5N1 detected in birds sampled at TRC during 2022 represented the same genotypes detected in wild birds sampled within the Mississippi flyway, confirming that during active disease outbreaks wildlife rehabilitation centers are viable resources to augment wider surveillance efforts^9^.

A limitation with extrapolation of these results to a larger population is the lack of HP H5N1 experimental data in raptor species. The details around infectious dose, severity of disease, and survival rate for HP H5N1 under controlled conditions are poorly understood in most raptor species as are the potential effects of existing immunity of these responses. A few experimental studies have been performed in raptor species however they were not performed with the currently circulating HP H5N1 strain or with the species described in this study limiting the ability to extrapolate^12,15,25,26^. While this paper provides evidence of raptors surviving infection with HP H5N1, it could not be determined when infections occurred or if surviving birds developed clinical disease. It also is unknown if there will be a subsequent decrease in severity of disease in birds attributable to this acquired immunity or how long such potential protection will last.

The results of this study are consistent with raptors surviving infections with HP H5N1. The data also are consistent with a higher rate of HP H5N1 infection in bald eagles than can be estimated from virologic testing of dead or moribund birds. There is a clear need for continued surveillance for both active HP H5N1 infections and seroprevalence in raptor species and additional research in order to better understand the effects and the potential trajectory of this outbreak in wild raptor populations.

## Methods

### Sample populations

TRC routinely admits approximately 1000 wild raptors per year from the state of Minnesota and surrounding states. Birds that are found injured, sick, or orphaned are brought to the center for medical care, most often by members of the public. HRBO is a bird banding and research station located in Duluth, Minnesota. HRBO bands nearly 3000 migratory raptors each fall^27^. Both TRC and HRBO receive birds from the Mississippi flyway^28^.

### Sample collection

Serum and/or plasma samples were obtained from wild raptors admitted to TRC that tested negative on RT-PCR for IAV between September 13, 2022, and April 27, 2023. This time frame encompasses both fall and spring avian migrations in the northern portion of the Mississippi flyway^28^. Both serum and plasma were concurrently drawn on 73 patients early in the study to evaluate concordance between sample types. After March 30, 2023, only plasma was drawn on TRC patients. Samples were collected on admission, or as soon as possible after admission if the patient was too medically unstable to obtain blood. Strict biosecurity measures were in place and no in-hospital HP H5N1 viral transmission was detected via active surveillance during the study period^9^.

At HRBO, serum samples were collected opportunistically between September 28, 2022, and November 18, 2022, in conjunction with their annual banding efforts. Birds were captured, sampled, banded, and released all within the same day. All birds appeared outwardly healthy and paired swab samples for RT-PCR were not obtained.

Blood was collected using standard venipuncture techniques. Total blood volume collected did not exceed 1% of body weight. To obtain serum, immediately following collection the blood was placed into 1.5 mL polypropylene microcentrifuge tubes and allowed to clot. Blood for plasma was collected using a pre-heparinized syringe and needle, and then transferred into 1.5 mL polypropylene microcentrifuge tubes. Blood was centrifuged at 2000 g for 10 min. Serum or plasma was then pipetted into microcentrifuge tubes and stored frozen at −20 °C until analysis.

### Serologic testing

Raptors included in this study were tested for exposure to H5 and N1 subtypes of IAV through a two-step approach. Serum or plasma samples were initially tested for antibodies to the internal nucleoprotein (NP) of IAV. These antibodies indicate exposure to IAV regardless of subtype or pathotype. The goal of this initial screening was to identify samples that were candidates for H5 and N1 serologic testing. Any samples seropositive for IAV NP antibodies were subsequently tested for antibodies to the H5 and N1 subtypes.

### General screen for avian influenza antibodies

Prior to serologic testing, serum and plasma samples were heat inactivated at 56 °C for 30 min. Serum and/or plasma samples were tested for the presence of IAV NP antibodies using a commercial blocking enzyme-linked immunosorbent assay (bELISA, AI MultiS-Screen Ab Test, IDEXX Laboratories, Westbrook, Maine, USA). The bELISA testing was performed in accordance with the manufacturer’s directions. Based on the manufacturer’s directions, samples with a mean sample absorbance to negative control (S/N) value < 0.5 are positive for IAV NP antibodies. To increase the sensitivity of this serologic screening tool, we considered any samples with a mean S/N value < 0.7 as positive for IAV NP antibodies, and eligible for H5 and N1 serologic testing. This revised diagnostic threshold was determined from previous H5 and N1 serologic studies conducted in waterfowl^29,30^.

### Testing for antibodies to H5 and N1

Serum or plasma samples that tested positive for IAV antibodies on the commercial bELISA were subsequently tested for H5 and N1 antibodies using hemagglutination inhibition (HI) assay, virus neutralization (VN), and enzyme linked lectin binding assay (ELLA), using previously described methods^23,31^. Two reverse genetic (rg) antigens representing clade 2.3.4.4b H5 HP IAV (IDCDC-RG71A) and North American low pathogenic (LP) H5 (rgBWT) were used in HI tests. For VN, only the representative clade 2.3.4.4b antigen was used. The LP rgBWT (H5N2) antigen includes the hemagglutinin (HA) and neuraminidase (NA) from A/blue-winged teal/Texas/AI12-4150/2012 on a A/Puerto Rico/8/1934 (PR8) backbone and IDCDC-RG71A (H5N8) includes the HA and NA from A/Astrakhan/3212/2020-like virus on a PR-8 backbone. The HA of IDCDC-RG71A contains a modified protease cleavage site characteristic of LP IAV. For HI and VN, titers of ≥ 32 and ≥ 20 were considered positive for antibodies to H5, respectively. For ELLA, A/ruddy turnstone/New Jersey/AI13-2948/2013 (H10N1) was used as an antigen and a titer of ≥ 80 was regarded as positive for antibodies to N1. For each raptor species tested, a minimum of five bELISA negative sera from the same species were also tested as negative controls. A bird was considered positive for H5 or N1 antibodies if it tested positive in any of the H5 assays or ELLA, respectively.

### Sample population characteristics

Presumed sex and age was determined based on weight, size, and plumage compared to established species reference ranges for the upper Midwest^32–36^. All species except for eagles were categorized as adults if they were aged as second year or older, or as juvenile birds if they had hatched within the same calendar year of admission^35,36^. Bald eagles were aged as adults if they had definitive adult plumage, subadults if they were aged as between their second and fifth year, and as juveniles if they hatched within the same calendar year of admission^37^.

### Statistical analysis

Results from serum and plasma samples obtained from the same individual at the same point in time were compared using a paired sample T test (Microsoft Excel).

## Data Availability

Data from this study are available from the corresponding author upon request.
